# Supplementary material for: Complexome profiling of the Chlamydomonas psb28 mutant reveals TEF5 as an early PSII assembly factor
Source: Plant Cell. 2025 Mar 17;37(6):koaf055. doi: 10.1093/plcell/koaf055 (PMC12164590; doi:10.1093/plcell/koaf055)
Supplement: koaf055_Supplementary_Data [file koaf055_supplementary_data.zip › psb28_interactive.zip!/plots/intensity/qualityPlots/peptides/NSS4_Cre17.g721450.t1.1.html]

### Cre17.g721450.t1.1

Trivial name: NSS4  
SeqCoverage [median]: 5.90  
Mass: 63.07 kDa  
  
 

| Sequence | MissedCleavage | Mass [Da] | wtIntensity | muIntensity | Score |
| --- | --- | --- | --- | --- | --- |
| FDLLSSPR | 0 | 933.49 | 0.00 | 1043.37 | 60.59 |
| GPGISGVLR | 0 | 854.50 | 0.00 | 1771.28 | 98.52 |
| LGDLDAKGDTAYTAMSS | 1 | 1714.77 | 0.00 | 414.10 | 71.81 |
|  |  |  | 0.0 | 3228.7 |  |
